# Whole-genome comparison between reference sequences and oyster *Vibrio vulnificus* C-genotype strains

**DOI:** 10.1371/journal.pone.0220385

**Published:** 2019-07-30

**Authors:** Abraham Guerrero, Alexei Fedorovish Licea-Navarro, Ricardo González-Sánchez, Marcial Leonardo Lizárraga-Partida

**Affiliations:** Centro de Investigación Científica y de Educación Superior de Ensenada Baja California, México, CICESE, Ensenada Baja California, México; Wilfrid Laurier University, CANADA

## Abstract

Whole-genome sequences of *Vibrio vulnificus* clinical genotype (C-genotype) from the CICESE Culture Collection, isolated from oysters, were compared with reference sequences of CMCP6 and YJ016 *V*. *vulnificus* C-genotype strains of clinical origin. The RAST web server estimated the whole genome to be ~4.8 Mb in CICESE strain 316 and ~4.7 Mb in CICESE strain 325. No plasmids were detected in the CICESE strains. Based on a phylogenetic tree that was constructed with the whole-genome results, we observed high similarity between the reference sequences and oyster C-genotype isolates and a sharp contrast with environmental genotype (E-genotype) reference sequences, indicating that the differences between the C- and E-genotypes do not necessarily correspond to their isolation origin. The CICESE strains share 3488 genes (63.2%) with the YJ016 strain and 3500 genes (63.9%) with the CMCP6 strain. A total of 237 pathogenicity associated genes were selected from reference clinical strains, where—92 genes were from CMCP6, 126 genes from YJ016, and 19 from MO6-24/O; the presence or absence of these genes was recorded for the CICESE strains. Of the 92 genes that were selected for CMCP6, 67 were present in both CICESE strains, as were as 86 of the 126 YJ016 genes and 13 of the 19 MO6-24/O genes. The detection of elements that are related to virulence in CICESE strains—such as the RTX gene cluster, *vvhA* and *vvpE*, the type IV pili cluster, the XII genomic island, and the *viuB* genes, suggests that environmental isolates with the C-genotype, have significant potential for infection.

## Introduction

*Vibrio vulnificus* is an acute human pathogen that is commonly isolated from seafood and warm estuarine waters, where their growth is affected by temperature and salinity [1]. *V*. *vulnificus* has been isolated in the US [2], France [3], Israel [4], China [5], and Taiwan [6]. In Mexico, although the isolation of *V*. *vulnificus* has been reported [7–9], the Mexican health system has not registered any clinical case.

According to the FAO (Food and Drug Administration) and the WHO (World Health Organization) [10], the virulence in *V*. *vulnificus* strains appears to be multifaceted and is poorly understood. Thus, all *V*. *vulnificus* should be considered virulent. Some virulence factors, that were positive correlated with virulence, are present in nearly all *V*. *vulnificus* strains that are isolated from clinical cases or the environment, such as capsular polysaccharides [11], or cytolysin-hemolysin (VvhA), which induces acute cell death and is important in the pathogenesis and dissemination of these bacteria [12]. Nevertheless, its frequent isolation in the environment does not necessarily correlate with a high number of clinical cases, indicating that not all strains are equally virulent [13].

*V*. *vulnificus* causes gastroenteritis, necrotizing infections, and acute primary septicemia [14], the latter of which results in a high mortality rate, primarily in individuals with chronic liver disease or immunodeficiency disorders [15–17]. Contaminated water and raw or undercooked seafood, especially oysters, are vectors of this infection [14]. *V*. *vulnificus* infections are a major cause of mortality that is associated with seafood-borne diseases in the US, reaching a mortality rate of 95% [18].

Based on several genetic analyses in *V*. *vulnificus*—such as the virulence correlated gene (*vcg*) [19], multilocus sequence typing (MLST) [20], 16S rRNA [21], sequencing of multifunctional autoprocessing RTX toxin (MARTXVv) [22], and whole-genome analysis [23],—there are 2 genotypes: environmental and clinical. Most environmental isolates are defined as the environmental (E-) genotype, whereas strains that are isolated from human infections are predominately the clinical (C-) genotype.

Different authors [19, 21, 24], have suggested that strains with the E-genotype are less virulent than those with the C-genotype. Yet, Thiaville et al. [25] found that greater virulence in *V*. *vulnificus* correlated with the clinical genotype but not exclusively. Kwak et al. [22], reported that the MARTX_Vv_ toxin is linked to the ability of *V*. *vulnificus* to cause disease and proposed 2 MLST lineages for *V*. *vulnificus* strains, of which lineage I, defined as the virulence-conferring lineage, contained strains of human origin.

On sequencing 3 strains of *V*. *vulnificus* with the E-genotype and comparing them with reference sequences of 3 C-genotype strains, Morrison et al. [23], identified 278 genes that differentiated clinical and environmental genotypes. But, they did not include an environmental isolate with a C-genotype. Whole-genome analyses of *V*. *vulnificus* have focused on C-genotype strains from clinical samples, resulting in a lack of studies on C-genotype strains from environmental sources. Using the MLST and *rtxA1* approaches, Guerrero et al. [8], noted high genetic similarity between C-genotype strains from clinical cases and environmental samples and sharp differences with E-genotype strains.

Because specific pathogenic factors that differentiate between high- and low-virulence *V*. *vulnificus* strains have not been established, whole-genome analysis could increase our understanding of the differences between clinical and environmental isolates within the same genotype. This study compared high-virulence C-genotype reference sequences with C-genotype strains isolated from oysters studied by Guerrero et al. [8], to confirm the absence or presence of specific pathogenic genes, in C-genotype strains from environmental sources.

## Material and methods

We have compared the whole-genome sequences of 2 *V*. *vulnificus* strains from oyster samples that have been studied by Guerrero et al. [8]—CICESE-316 and CICESE-325 (MLST lineage I, C-genotype, *rtxA1*-C type)—with 2 reference genomes of the *V*. *vulnificus* strains CMCP6 and YJ016 (MLST lineage I, C-genotype, *rtxA1*-C type), which have been reported to be acute human pathogens (Genbank Accession Numbers AE016795.3, AE016796.2, and BA000037.2, BA000038.2, respectively) and examined by several groups [22, 23, 26–28].

### Genome sequencing

A single colony of the CICESE strains was used to inoculate Zobell´s marine broth and was grown overnight at 35°C. Genomic DNA was extracted from the cultures with the Wizard Genomics^TM^ DNA Purification Kit (Promega, Madison, WI, USA), according to the manufacturer’s instructions. The genomic DNA was sequenced (paired-end) on a Miseq^TM^ platform (Illumina Inc., USA).

Sequenced reads of the CICESE strains were mapped to the two chromosomes of the reference genome YJ016, using BWA-MEM VO.7.12 [29], with default parameters. The mapping statistics were extracted with SAMtools VO1.2 [30]. For each genome, the coverage depth was calculated using Qualimap V2.2.1 [31]. The reads were assembled using VAGUE V1.0.5 [32] and CAP3 [33].

The contigs that were obtained from the CICESE strains were submitted to the Rapid Annotation Using Subsystem Technology (RAST) [34] and PathoSystems Resource Integration Center (PATRIC) web servers [35], to determine the annotation of the genes.

Because we focused on determining the presence or absence of C-genotype and pathogenesis-related genes, as reported by Chen et al. [26], Gulig et al. [36], and Morrison et al. [23], each gene from the CMCP6, YJ016, and MO6-24/O, was evaluated by alignment with the annotated contigs that were generated for the CICESE strains using the RAST and PATRIC web servers.

Original sequences were submitted to GenBank, with the accession numbers QKYO00000000 for *V*. *vulnificus* CICESE-316 and QKYP00000000 for *V*. *vulnifucus* CICESE-325, (BioProject: PRJNA475608).

### Genomic comparison

**Fig 1:**
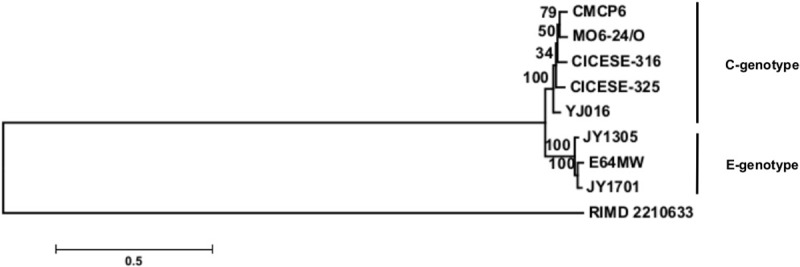
Phylogenetic tree of *V*. *vulnificus* C-genotype and E-genotype strains, using *V*. *parahaemolyticus* RIMD 2210633 genome as out-group. Bootstrap values for each node are indicated in the tree. Analysis was implemented with SNP using the maximum likelihood method (Kimura 2-parameters model), with 1000 bootstraps replicates.

A genotype phylogenetic tree was constructed with whole-genome data from 2 C-genotype CICESE strains and data from Morrison et al. [23] on 3 C-genotype reference genomes (CMCP6, YJ016, MO6-24/O) and 3 E-genotype strains (JY1305, E64MW, JY1701), as well as from RIMD 2210633 *V*. *parahaemolyticus* strain, used as an outgroup. The genomes were fist compared with Parsnp V1.2 in the Harvest suite V1.1.2 [37] to detect single-nucleotide polymorphisms (SNPs) among the compared genomes. The obtained sequences were used to generate the phylogenetic tree (Fig 1) implemented in MEGA V6.06 [38], using the maximum composite likelihood method (Kimura 2-parameter model), with 1000 replicates for bootstrapping.

The number of common genes between the CICESE genomes and the YJ016 and CMCP6 reference sequences was evaluated using PATRIC web server annotations, and then compared with the Venny web application (http://bioinfogp.cnb.csic.es/tools/venny/index.html).

Supercontigs were implemented for each CICESE strain in MeDuSa scaffolder [39] (http://combo.dbe.unifi.it/medusa) using the YJ016 strain as a reference genome. Supercontigs for chromosomes I and II, were used to perform a whole-genome comparison, implemented in Blast Ring Image Generator (BRIG, V0.95) [40].

## Results

Sequencing of CICESE-316 and CICESE-325 resulted in 2,117,568 and 3,238,599 (2x ~140 bp) paired-end reads, with N50 = 14,356 and 20,259 respectively. The depth of sequencing coverage was equivalent to 50.52x and 73.35x, with a GC content of 46.9% and 46.8%; the sequences were assembled in 847 and 677 contigs for CICESE-316 and CICESE-325 (Table 1). Using the RAST web server, the estimated size of the entire genome for these strains was ~4.76 and ~4.71 Mb. A total of 4,234 and 4,217 coding sequences (CDS) were detected for CICESE-316 and CICESE-325, respectively as well as 13 and 15 RNAs. No plasmids were detected in the CICESE strains (Table 1).

**Table 1:** Summary of assembly and annotation characteristics of CICESE-316 and CICESE-325 genomes. GC%: Guanine+Cytocine content in chromosomes. CDS: Coding sequences. RNAs: Ribonucleic acids. N50: Minimum contig length needed to cover 50% of the genome. L50: Number of contigs whose length sum makes up 50% of the genome size.

	CICESE-316	CICESE-325
Genome Size	4,762,000	4,715,706
Reads (2x ~140)	2,117,568	3,238,599
Contigs	847	677
Coverage Depth	50.52x	73.35x
GC%	46.9	46.8
CDS	4,234	4,217
RNAs	13	15
N50	14,356	20,259
L50	107	76

The phylogenetic tree in Fig 1 shows the association between the CICESE strains and C-genotype reference genomes (CMCP6, YJ016, and MO6-24/O) and a clear separation from those with the E-genotype (E64MW, JY1305 and JY1701).

**Fig 2:**
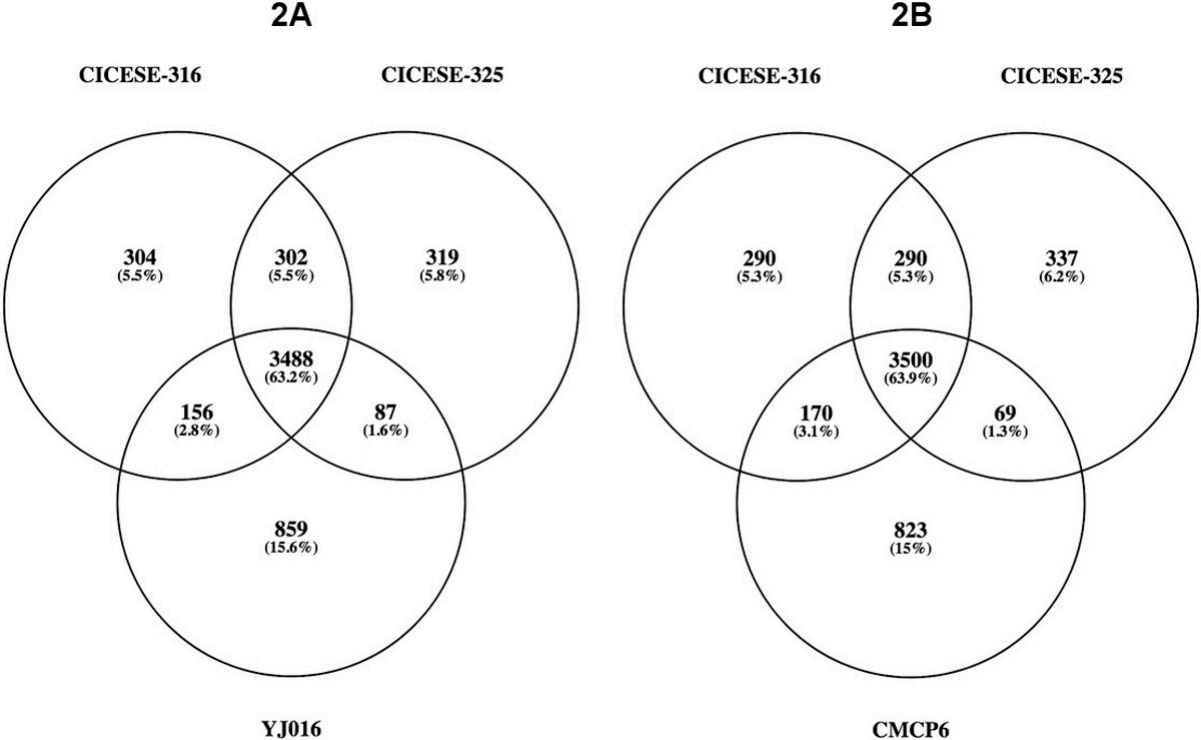
Venn diagram representing not-shared and common genes among CICESE-316 and CICESE-325, related to YJ016 (2A) and CMCP6 (2B).

The Venn diagrams in Fig 2A and 2B shows the number of not-shared and common genes between CICESE genomes and the YJ016 (Fig 2A) and CMCP6 (Fig 2B) reference sequences. Strain YJ016 shared 3488 genes (63.2%) with both CICESE strains, and CMCP6 shared 3500 genes (63.9%), also with both CICESE strains. CICESE-316 and CICESE-325 share respectively 156 (2.8%) and 87 genes (1.6%) with YJ016 and 170 (3.1%) and 69 genes (1.3%). with CMCP6. CICESE-316 and CICESE-325 had 304 and 319 unique genes, respectively, that were not present in YJ016 (Fig 2A), versus 290 and 337 unique genes that were not recorded in CMCP6 (Fig 2B). YJ016 had 859 genes (15.6%) and CMCP6 had 823 genes (15%) that were not detected in either CICESE strain.

The CDS that we obtained were classified into 26 categories (RAST web server). Categories that were related to virulence, disease, and defense were enriched in 88 and 83 CDS for CICESE-316 and CICESE-325, respectively, whereas resistance to antibiotics and toxic compounds were represented in 70 and 66 CDS.

Of the 52 genes that were reported by Morrison et al. [23] and present only in C-genotype strains, all were detected in CICESE-316, compared with 33 in CICESE-325. Further, the corresponding virulence genes to cytotoxin, hemolysin, and RTX toxin (VVA0964, VVA0965, VV0508, VV0601, VV0795, VV0914, VV1495, VV2791, VV3230, VVA0118, VVA0303, VVA1339 and VVA1030), as reported by Chen et al. [26], were found in both strains.

Table 2 presents a select list of genes that were exclusive to *V*. *vulnificus* C-genotype pathogenic strains (CMCP6, YJ016, and MO6-24/O), based on Chen et al. [26], Gulig et al. [36], and Morrison et al. [23]. From this list, 237 genes were related to pathogenic C-genotype strains, with 92 genes corresponding to the CMCP6 genome, 126 genes corresponding to the YJ016 genome, and 19 corresponding to the MO6-24/O genome. CICESE-316 had 205/237 positive matches and CICESE-325 had 166/237 positive matches with respect to the 3 reference strains. A total of 67 of 92 genes for CMCP6 were present in both CICESE strains, versus 86 of 126 for YJ016 and 13 of 19 for MO6-24/O (Table 2).

**Table 2:** List of pathogenicity C-genotype genes of *V*. *vulnificus* reported for pathogenic strains, CMCP6, MO6-24/O and YJ016, after Gulig et al. [36], Chen et al. [26] and Morrison et al. [23].

CMCP6	CICESE-316	CICESE-325	CMCP6	CICESE-316	CICESE-325	YJ016	CICESE-316	CICESE-325
VV1_0456	+	+	VV2_1203	-	+	VV3176	-	-
VV1_0457	+	+	VV2_1204	-	+	VV3230	+	+
VV1_0458	+	+	VV2_1273	-	-	VVA0118	+	+
VV1_0459	+	+	VV2_1274	-	-	VVA0202	+	+
VV1_0465	+	+	VV2_1275	-	-	VVA0303	+	+
VV1_0515	+	+	VV2_1290	+	+	VVA0325	+	-
VV1_0776	+	-	VV2_1303	+	+	VVA0326	+	-
VV1_0789	-	-	VV2_1304	+	+	VVA0327	+	-
VV1_1090	+	-	VV2_1309	+	+	VVA0329	+	-
VV1_1094	+	+	VV2_1363	+	+	VVA0331	+	-
VV1_1095	+	+	VV2_1509	+	-	VVA0332	+	-
VV1_1518	+	+	VV2_1510	+	-	VVA0333	+	-
VV1_1751	+	+	MO6-24/0			VVA0362	+	+
VV1_2031	-	-	VVMO6_02633	+	+	VVA0389	+	-
VV1_2037	-	-	VVMO6_02634	+	+	VVA0390	+	-
VV1_2038	-	-	VVMO6_02635	+	+	VVA0392	+	-
VV1_2061	+	+	VVMO6_03282	+	-	VVA0393	+	-
VV1_2114	-	-	VVMO6_03283	+	-	VVA0395	+	-
VV1_2115	-	-	VVMO6_03523	+	-	VVA0419	+	+
VV1_2158	+	+	VVMO6_03524	+	-	VVA0420	+	+
VV1_2183	-	-	VVMO6_03525	+	-	VVA0421	+	+
VV1_2184	-	-	VVMO6_03526	+	-	VVA0422	+	+
VV1_2228	+	+	VVMO6_04101	+	+	VVA0423	+	+
VV1_2321	+	+	VVMO6_04102	+	+	VVA0424	+	+
VV1_2326	+	+	VVMO6_04103	+	+	VVA0509	+	+
VV1_2327	+	+	VVMO6_04104	+	+	VVA0510	+	+
VV1_2329	+	+	VVMO6_04105	+	+	VVA0511	+	+
VV1_2330	+	+	VVMO6_04106	+	+	VVA0581	+	-
VV1_2331	+	+	VVMO6_04498	+	+	VVA0582	+	-
VV1_2332	+	+	VVMO6_04499	+	+	VVA0583	+	-
VV1_2333	+	+	VVMO6_04500	+	+	VVA0584	+	-
VV1_2334	+	+	VVMO6_04501	+	+	VVA0618	+	+
VV1_2335	+	+	YJ016			VVA0619	+	+
VV1_2336	+	+	VV0300	-	-	VVA0620	+	+
VV1_2337	+	+	VV0301	-	-	VVA0781	-	+
VV1_2338	+	+	VV0302	-	-	VVA0782	+	+
VV1_2339	+	+	VV0303	-	-	VVA0916	+	+
VV1_2340	+	+	VV0309	+	-	VVA0917	+	+
VV1_2341	+	+	VV0337	+	-	VVA0918	+	+
VV1_2401	+	+	VV0339	+	-	VVA0964	+	+
VV1_2708	+	+	VV0340	+	-	VVA0965	+	+
VV1_2748	+	+	VV0361	-	-	VVA1024	+	+
VV1_2758	+	+	VV0508	+	+	VVA1025	+	+
VV1_2840	+	+	VV0601	+	+	VVA1026	+	+
VV1_2868	+	+	VV0795	+	+	VVA1029	+	+
VV1_3144	-	+	VV0914	+	+	VVA1030	+	+
VV2_0019	+	+	VV1465	+	+	VVA1032	+	+
VV2_0073	+	-	VV1491	+	+	VVA1034	+	+
VV2_0074	+	-	VV1495	+	+	VVA1035	+	+
VV2_0075	+	-	VV1546	-	-	VVA1036	+	+
VV2_0076	+	-	VV1605	+	+	VVA1037	+	+
VV2_0077	+	-	VV1615	+	+	VVA1113	+	+
VV2_0078	+	-	VV1631	+	+	VVA1115	+	+
VV2_0212	+	+	VV1738	-	-	VVA1116	+	+
VV2_0313	+	+	VV1754	-	-	VVA1199	+	+
VV2_0627	-	-	VV1767	+	+	VVA1200	+	+
VV2_0726	+	+	VV1774	+	-	VVA1201	+	+
VV2_0729	+	+	VV1775	+	-	VVA1202	+	+
VV2_0730	+	+	VV1786	-	-	VVA1294	+	+
VV2_0731	+	+	VV1791	+	+	VVA1295	+	+
VV2_0732	+	+	VV1806	+	+	VVA1297	+	+
VV2_0733	+	+	VV1812	+	+	VVA1299	+	+
VV2_0735	+	+	VV1818	-	-	VVA1300	+	+
VV2_0782	+	+	VV1831	-	+	VVA1301	+	+
VV2_0783	+	+	VV1842	-	-	VVA1303	+	+
VV2_0851	+	+	VV1852	-	+	VVA1304	+	+
VV2_0864	+	+	VV1854	+	+	VVA1306	+	+
VV2_0868	+	+	VV2040	+	+	VVA1308	+	+
VV2_0881	+	+	VV2041	+	+	VVA1309	+	+
VV2_0884	+	+	VV2043	+	+	VVA1310	+	+
VV2_0993	+	+	VV2191	-	-	VVA1339	+	+
VV2_0994	+	+	VV2778	+	-	VVA1413	-	-
VV2_1075	+	+	VV2779	+	+	VVA1504	+	+
VV2_1106	+	+	VV2780	+	+	VVA1505	+	+
VV2_1107	+	+	VV2781	+	+	VVA1506	+	+
VV2_1108	+	+	VV2791	+	+	VVA1632	+	+
VV2_1109	+	+	VV2872	+	+	VVA1633	+	+
VV2_1138	+	+	VV2874	+	+	VVA1634	+	+
VV2_1149	+	+	VV2990	+	+	VVA1635	+	+
VV2_1186	+	+	VV3118	+	+			

**Fig 3:**
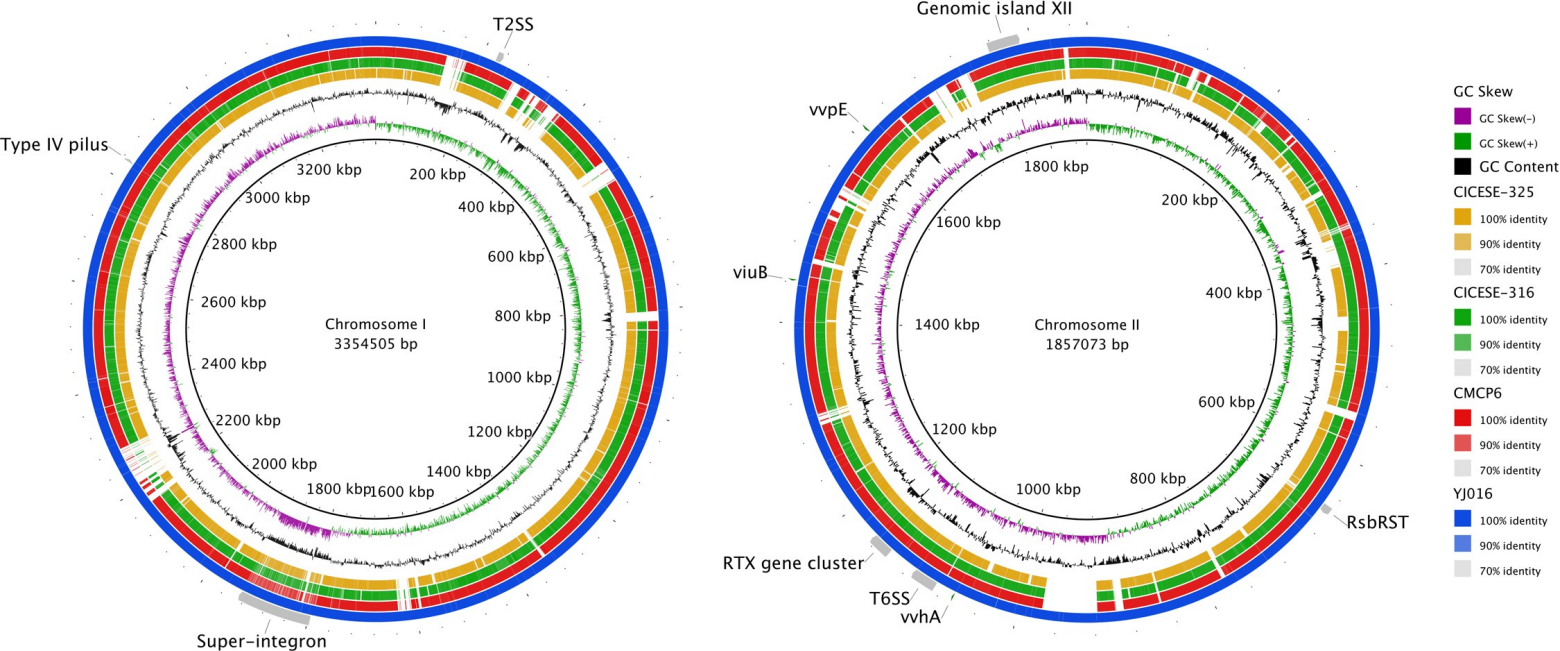
Circular maps alignment of *V*. *vulnificus* genomes CICESE-316, CICESE-325 and CMCP6, and YJ016 as reference genome, implemented with BRIG V0.95.

Sequences were assembled for the 2 chromosomes for the CICESE strains and CMCP6 and compared with the reference genome of YJ016. Compararison show gaps in different zones in both chromosomes (Fig 3). The most notorious gaps for CICESE strains and CMCP6 were found between 200 to around 2250 kpb in the chromosome I. Chromosome II showed several differences among CICESE strains and CMCP6 with YJ016, most notably at 920 kbp. A region with low GC content was also detected in chromosome I, located between 1750 and 1950 kpb (Fig 3) corresponding to a super-integron (SI). This region has high homology with a genomic SI region on chromosome I of YJ016 (VV1745 to VV1941).

Three secretions systems were also detected in both CICESE strains: T1SS, T2SS, and T6SS. The genomic island XII, located on the small chromosome, was also detected in both CICESE strains, with >49.8% GC content and differences in nucleotides of 1.8% or less (477 SNP in CICESE-316 and 586 SNP in CICESE-325) with respect to nucleotides reported for YJ016 in genomic island XII. The *viuB* gene was detected in both strains, but the *rsbRST* operon was only present in CICESE-316.

## Discussion

The assembly and annotation of the CICESE strains (Table 1) were similar to those for the *V*. *vulnificus* reference genomes. The estimated sizes for the entire CICESE-316 and CICESE-325 genomes were ~4.76 and ~4.71 Mb, respectively—smaller than the 5.2 Mb that has been reported for CMCP6 and FORC_017, the 5.3-Mb YJ016 genome, and the 5.0-Mb MO6-24/O genome [26, 27, 41]. These data show that the differences between C-genotype *V*. *vulnificus* genomes are within ~0.3 Mb. Plasmids were absent from the CICESE strains, as in the strains CMCP6 and MO6-24/O, in contrast to YJ016 and FORC_017, in which the presence of a plasmid has been reported.

The phylogenetic analysis in Fig 1 shows that select C-genotype reference genomes (CMCP6, YJ016, MO6-24/O) grouped with the C-genotype CICESE strains and differed sharply from the E-genotype reference genomes (JY1305, E64MW, JY1701). These whole-genome results confirm that the C-genotype cluster of *V*. *vulnificus* strains does not necessarily correspond to their clinical or environmental isolation. Guerrero et al. [8], have reported close homology between environmental isolates of C-genotype strains by PFGE, MLST, and *rtxA1* analysis, in which *V*. *vulnificus* C-genotype strains from oysters clustered with C-type *rtxA1 V*. *vulnificus* reference strains from clinical cases (CMCP6 or YJ016), within MLST virulence lineage I, as proposed by Kwak et al. [22].

Of the shared and not-shared genes between the 2 reference C-genotype *V*. *vulnificus* strains, as reported by Morrison et al. [23], YJ016 has 777 and CMCP6 has 332 genes that are exclusive to these reference strains, similar to the number of not-shared genes (Fig 2A and 2B) for CICESE-316 and CICESE-325 (between 290 to 337 genes). These findings indicate that the quantity of not-shared genes recorded in the CICESE and reference strains is normally reported in comparative genomic analyses of C-genotype strains.

Most of the pathogen-related genes associated to pathogenic strains with C-genotype that were identified by Chen et al. [26], Gulig et al. [36], and Morrison et al. [23] for the CMCP6, YJ016, and MO6-24/O strains were detected in the CICESE strains. Conversely, 12 and 14 pathogen-related genes that have been reported for CMCP6 and YJ016, respectively, were absent from both CICESE strains (Table 2).

Most groups of genes that are related to pathogenicity clusters [26] were detected in the CICESE strains. In the alignment with the YJ016 chromosomes (Fig 3), the CICESE and CMCP6 sequences had nearly the same differences in both chromosomes with respect to YJ016. According to Thiaville et al. [25], both reference strains show high virulence for skin and liver infections and have similar lethality; thus, the differences between CMCP6 and CICESE strains, with YJ016, might not be essential for virulence.

The RTX gene cluster; the *vvhA* and *vvpE* genes; the secretion systems T1SS, T2SS, and T6SS; the *viuB* gene; the type IV pili cluster; and the genomic island XII—all of which were detected in the CICESE strains—have been identified as important virulence factors.

### RTX gene cluster

The CICESE strains harbored the RTX gene cluster, with significant identity as the *rxtA1* gene in the CMCP6 reference genome (Fig 3). The RTX gene cluster (~22.5 kbp), located on the small chromosome, includes *rtxA1* (VVA1030), which encodes the MARTXvv toxin, and *rtxC* (VVA1032), *rtxB* (VVA1034), *rtxD* (VVA1035), and *rtxE* (VVA1036) in YJ016 (Table 2), as reported by Chen et al. (2003). The gene rtxA1 (VVA1030) that encode the repeats-in-toxin (RTX) exoprotein, is considerate an important virulent factor in *V*. *vulnificus* [22].

The MARTXvv toxin has been described as the main virulence factor *of V*. *vulnificus*; this toxin is involved in apoptosis and necrosis and is essential in the early stages of infection and its dissemination to the bloodstream [42–44]. Partial or total deletion of *rtxA1*, decrease its cytotoxicity and ability to infect and results in a 2600-fold increase in its LD_50_ in an animal model [22,42,43,45].

The *rtxB*, *rtxD*, *rtxE*, and *tolC* genes encode for structural proteins in the type I secretion system (T1SS), which mediates the release of the MARTX toxin to the surrounding environment [46,47]. The deletion of *rtxE* affects the secretion of MARTX, and mutant strains have lower cytotoxic activity in cell lines [48].

### *vvhA* and *vvpE*

The *vvhA* and *vvpE* genes, which have been reported in all *V*. *vulnificus* strains, were detected in the CICESE strains. *vvhA* (VV2_0404, CMCP6), an extracellular hemolysin, and *vvpE* (VV2_0032, CMCP6), a metalloprotease, have been implicated in necrosis, vascular permeability, apoptosis, pore formation, and tissue damage [49]. The secretion of *vvhA* and *vvpE* into the environment, is mediated by the type II (T2SS) secretion system [50], which was detected in both CICESE strains.

### Type IV pili cluster

The CICESE strains contained the type IV pilus gene cluster (*pilA*, *pilB*, *pilC*, and *pilD*; Fig 3). *pilA* variated significantly within VV2778, but few variations were found in VV2779, VV2780, and VV2781 compared with the YJ016 strain. *pilA* has been implicated in the adherence to host cells, biofilm formation, and virulence [51]. Chattopadhyay et al. [52], have suggested that the variability in *pilA* in *V*. *vulnificus* is associated with several functions, allowing it to adapt to various hosts. Therefore, these differences could be associated with the isolation of CICESE strains from oyster, compared with the clinical origin of reference strains.

### Genomic island XII

The genomic island XII, located on the small chromosome (VVA1613 to VVA1636) [53], was present in both CICESE strains, with few differences compared with YJ016 (Fig 3). This 33-kb region, which has an aberrant GC content of 50%, correlates with high-virulence C-genotype strains and confers a selective advantage in the environment or human host [53]. Morrison et al. [23], reported that this region is present in C-genotypes but not in E-genotypes. This region harbors 2 chondroitinase genes, an ABC transport system, the arylsulfatase A gene cluster, and hypothetical proteins [53]. The arylsulfatase (*aslA*) gene has been implicated in the invasion of the blood-brain barrier in *E*. *coli* [54], and chondroitinase has been described as a virulent factor in certain fungi, such as *Paracoccidioides brasiliensis* [55].

### Additional pathogenic-associated genes

Strains with the C-genotype are more resistant to stressful conditions than E-genotype strains [56]. The ability to survive under stressful conditions has been associated with the presence of the RsbRST stress module genes (*rsbR*, *rsbS*, *rsbT*, and *rsbU*) and the siderophore-encoding (*viuB*) gene [23,24]. Williams et al. [57] evaluated strains that contained the *rsbRST* operon and found it to be specific to C-genotypes, wherein 75% of C-genotypes and no E-genotypes harbored the entire operon. Bogard and Oliver [24], have reported that in the C-genotype strains that they studied, they detected the *viuB* gene, whereas few E-genotype strains were positive for this gene. The presence of both elements—*viuB* and the *rsbRST* operon—in CICESE-316 (Fig 3), indicates a greater ability to survive under stressful conditions.

The T1SS, T2SS, T4SS, and T6SS secretion systems, has been identified in *V*. *vulnificus* [27]. These systems are involved in the secretion of proteins, such as toxins, and the transport of DNA [58]. In addition to T1SS and T2SS, most of the genes associated with T6SS (VVA0970 to VVA0996, for YJ016), were also detected in CICESE strains (Fig 3).The genes associated to T4SS were not detected in the CICESE strains.

The region with low GC content between 1750 to 1950 Mb on chromosome I corresponds to the SI in the CICESE strains (Fig 3). This region is analogous to the genes that have been reported for YJ016 (VV1745 to VV1941) but differs from those of CMCP6 (VV1_2401 to VV1_2501), likely because the genes that are encoded within the SI are primarily strain-specific [36].

The CICESE strains that were isolated from oyster samples, showed high genomic similarity to reference C-genotype *V*. *vulnificus* strains. The detection of elements that are related to virulence—such as the *rxt* gene cluster, *vvhA* and *vvpE*, the type IV pili cluster, the genomic island XII, *viuB*, and the genes in Table 2—in the CICESE strains, suggests that environmental isolates with C-genotype, have a high potential for virulence and infection; this hypothesis should be tested in a future study on virulence.
